# Selection of High-Yielding and Stable Genotypes of Barley for the Cold Climate in Iran

**DOI:** 10.3390/plants12132410

**Published:** 2023-06-22

**Authors:** Alireza Pour-Aboughadareh, Habibollah Ghazvini, Seyed Shahriyar Jasemi, Solaiman Mohammadi, Sayed Alireza Razavi, Mehrdad Chaichi, Marefat Ghasemi Kalkhoran, Hassan Monirifar, Hamid Tajali, Asadollah Fathihafshjani, Jan Bocianowski

**Affiliations:** 1 Seed and Plant Improvement Institute, Agricultural Research, Education and Extension Organization (AREEO), Karaj P.O. Box 31587-77871, Iran; sh.jasemi@areoo.ac.ir; 2 Field and Horticultural Crops Research Department, Agricultural and Natural Resources Research and Education Center of West-Azarbayjan Province, Agricultural Research, Education and Extension Organization, Urmia P.O. Box 57169-63963, Iran; soleyman_45@yahoo.com; 3 Field and Horticultural Crops Research Department, Agricultural and Natural Resources Research and Education Center of Khorasan Razavi Province, Agricultural Research, Education and Extension Organization, Mashhad P.O. Box 91769-83641, Iran; a.razavi@areeo.ac.ir (S.A.R.); h.tajali1351@gmail.com (H.T.); 4 Field and Horticultural Crops Research Department, Agricultural and Natural Resources Research and Education Center of Hamedan Province, Agricultural Research, Education and Extension Organization, Hamedan P.O. Box 65199-91169, Iran; m.chaichi@areeo.ac.ir; 5 Field and Horticultural Crops Research Department, Agricultural and Natural Resources Research and Education Center of Ardabil (Moghan) Province, Agricultural Research, Education and Extension Organization, Ardabil P.O. Box 56951-57451, Iran; m.ardabil@gmail.com; 6 Crop and Horticultural Science Research Department, East Azarbaijan Agricultural and Natural Resources Research and Education Center, Agricultural Research, Education and Extension Organization (AREEO), Tabriz P.O. Box 51537-15898, Iran; monirifar@yahoo.com; 7 Field and Horticultural Crops Research Department, Agricultural and Natural Resources Research and Education Center of Markazi Province, Agricultural Research, Education and Extension Organization, Arak P.O. Box 38135-889, Iran; asad196747@yahoo.com; 8 Department of Mathematical and Statistical Methods, Poznań University of Life Sciences, Wojska Polskiego 28, 60-637 Poznań, Poland

**Keywords:** cold area, mega-environments, multi-environment trials, barley, stability analysis

## Abstract

The interaction between genotypes and environments plays an important role in selecting superior genotypes for target locations. The main objectives of the present study were to analyze the effect of the genotype-by-environment interaction (GEI) and identify superior, newly developed, and promising barley genotypes for cold regions in Iran. For these purposes, a set of genotypes obtained from breeding programs for cold climates in Iran, along with two reference genotypes, were investigated at eight research stations (Tabriz, Ardabil, Arak, Miandoab, Mashhad, Jolge Rokh, Karaj, and Hamadan) during two consecutive growing seasons (2019–2020 and 2020–2021). The results of the freezing test (LT_50_) showed that most of the tested genotypes had significant cold tolerance at the seedling stage. Based on the additive main effect and multiplicative interaction (AMMI) analysis, environment (E) and GEI effects explained 49.44% and 16.55% of the total variation in grain yield, respectively. Using AMMI1 and AMMI2 models, G2 and G20 were found to be superior genotypes in terms of grain yield and stability. Moreover, AMMI-based stability parameters considered the G20 genotype to be the ideal genotype. A two-plot analysis of the genotype-by-environment interaction (GGE) biplot showed that the 16 experimental environments were grouped into 2 mega-environments. Of the test environments, ARK1 and KAJ2 had the highest discriminating power and representativeness ability, and these were identified as ideal environments for testing advanced genotypes for yield and stability performance during early barley breeding practices in cold areas in Iran. In conclusion, both AMMI and GGE biplot models identified several superior genotypes, among which G20, with a high average yield relative to the overall average yield and the lowest IPC1 score, was found to have high yield stability and is recommended for inclusion in breeding programs for cold climates in Iran.

## 1. Introduction

Barley (*Hordeum vulgare* L.) is widely adapted to various environmental conditions. This cereal has a higher tolerance to environmental stresses such as salinity, drought, cold, etc. [1]. Barley is the fourth cereal crop in the world, and according to an FAO report, it was estimated that the average harvested area and global grain production would be ~504,000 hectares and 3.50 tons ha^−1^ in 2019, respectively (Faostat, http://www.fao.org/faostat/en/#home accessed on 1 December 2021). It was proven that barley kernels can be considered a wholesome food commodity, as they provide minerals, calcium and phosphorus, a moderate amount of protein and fiber, and B vitamins [2]. In Iran, and even in some developing countries, barley is known as the second most important food and feed crop after wheat [3]. Iran was one of the ten countries with the highest barley seed production in 2020 [4]. According to the latest statistics, in the 2020–2021 crop season, barley was grown on about 1.44 million hectares in the country, and its grain production was about 2.47 million tons [5]. The mountainous regions in Iran are considered one of the main barley growing centers, where low-temperature or cold stress limits barley production [6]. In these areas, soil temperatures can drop to near zero or even below zero [0 to −4 °C] in winter, which can severely damage seedlings. Therefore, screening barley germplasm for cold tolerance and identifying the most tolerant genotypes at an early stage of plant growth and development is a key breeding strategy for extending barley cultivation in the highlands and cold regions in Iran. Another important criterion for evaluating cold tolerance is to test the stability of grain yield for desirable genotypes in different locations with freezing winter temperatures [7].

As a general rule of thumb, accounting for the differential response of genotypes to environmental factors, especially climatic factors, has been one of the key challenges in plant breeding for decades. One of the major steps toward developing new varieties adapted to a wide range of environments is the evaluation of genotype–environment interactions (GEIs) [8,9]. When crop genotypes are evaluated in different environments (years, locations, and/or their combination), their yield performances can vary significantly [10]. The presence of a large GEI effect often leads to serious challenges for breeders when selecting high-yielding genotypes with overall adaptation [11]. Like other crops, barley is sensitive to environmental changes. According to Khalili and Pour-Aboughadareh [12], Vaezi et al. [13], Ahakpaz et al. [14], Hilmarsson et al. [15], Ghazvini et al. [6], and Nykiel et al. [16], barley yield is strongly affected by environmental changes. This means that GEI analysis plays a key role in commercial purposes and in identifying ideal target production environments to achieve maximum yield. Consequently, having a comprehensive knowledge of the GEI effect, measurements of adaptability and stability, and the influence of the genetic proportion of genotypes and environments on yield performance is essential for breeders when identifying the locations that should be used in their breeding programs [17].

Multi-environment trials (METs) are an important part of any breeding program aiming to identify and release high-yielding and stable genotypes for large-scale production in different environments, according to farmers’ preferences. However, the proper use of these trials requires an understanding of GEI effects [18]. There are many statistical tools for analyzing GEI and identifying stable high-yielding genotypes in different environments. Of all the methods, the additive main effect and multiplicative interaction (AMMI) analysis [19] and the genotype and genotype-by-environment interaction (GGE) biplot methodology [20] are the best models for analyzing such complex data sets. There are two main advantages of using the AMMI model: first, the model partitions the total variation into the main effects of genotypes and environments, as well as their interactions. Indeed, because of this flexibility, complex variation can be studied easily and separately. Second, it provides a pathway to predictive accuracy using the family members model [21]. Overall, the AMMI model allows breeders to use its valuable agricultural recommendation results to leverage both broad and narrow adaptations to increase productivity [22]. On the other hand, the GGE biplot model is a very useful tool and provides graphic images of MET data sets. This methodology is useful for (i) the selection of a high-performance genotype in a production environment, (ii) identifying the target environment for a specific genotype, (iii) determining the representativeness power and discriminating ability of test environments for further genotype assessments, (iv) deciphering the relationships between test environments, and (v) ranking the genotypes tested in term of both stability and yield performance [20]. Most GEI studies on barley have been performed in dry, temperate, and warm climates in Iran [1,12,13,14,23,24,25,26,27,28], and few were performed in cold regions. However, studies conducted in the cold climate in Iran have shown significant GEI in various regions [6,7] and supported the use of the AMMI analysis and the GGE biplot methodology for GEI analysis. With this in mind, the aims of this study were to (i) demonstrate the value of combining AMMI and GGE biplot models for GEI analysis and interpretation, (ii) identify actual mega-environments in cold regions in Iran, and (iii) assess the adaptability and stability of newly promising barley genotypes in different barley production environments.

## 2. Results

### 2.1. Response of Investigated Barley Genotypes to the Freezing Test

The results of the analysis of variance for LT_50_, thousand-grain weight, and grain yield are shown in Table 1. Accordingly, a significant difference was found between G4 and genotypes G12, G15, and G17 in terms of the LT_50_ indicator. On the contrary, differences between the tested genotypes were significant for thousand-grain weight and grain yield. LT_50_ ranged from −10 °C to −6 °C with an average of −7.58 °C, and among the genotypes tested, G4, with LT_50_ −10 °C, was the genotype with the best cold tolerance at the seedling stage. In addition, the thousand-grain weight ranged from 34.50 to 46.60 g with an average of 40.14 g. The highest values of this parameter were observed in genotypes G16, G17, and G19 (reference genotype). Grain yield showed high variability and ranged from 5.27 to 8.71 with an average of 7.13 tons ha^−1^. Genotype G1 (reference genotype), followed by G20 and G7, showed the highest grain yield compared to the other genotypes tested.

### 2.2. AMMI Analysis

**Figure 1:**
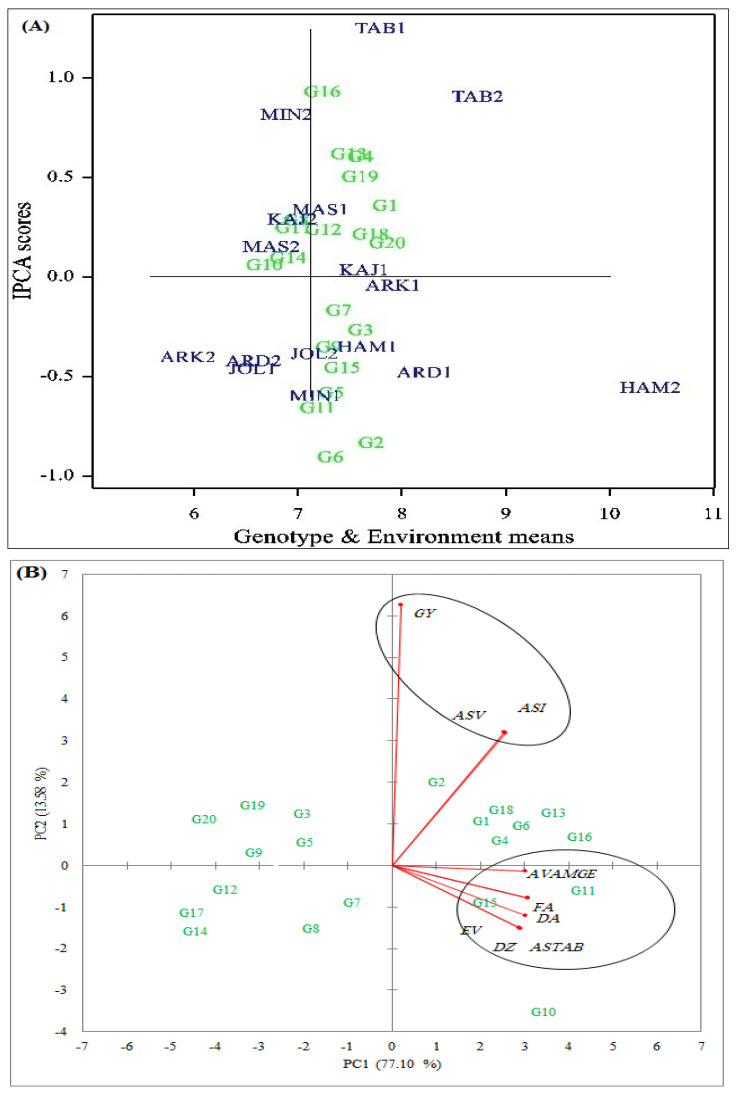
The AMMI1 biplot showing the GEI effect for 20 barley genotypes across 16 test environments in the cold climate in Iran (**A**). Grouping pattern of estimated AMMI-based stability parameters based on the first two components (**B**). GY, ASTAB, ASI, ASV, AVAMGE, DA, DZ, EV, and FA indicate grain yield, AMMI-based stability parameter, AMMI stability index, AMMI stability value, average sum across environments of the absolute value of the GEI modeled using AMMI, distance between the IPCA points and the origin in space (A and Z), average of the squared eigenvector values, and fitted AMMI model, respectively.

The result of the AMMI analysis showed that the effects of genotype (G), environment (E), and their interaction were highly significant (Table 2). The main effect of E accounted for 49.44% of the total variation, while G and GEI effects explained 4.99% and 16.55% of the variation, respectively. The GEI effect was divided into five significant IPCAs, each of which explained 22.98%, 18.20%, 13.48%, 11.49%, and 9.19%, respectively. AMMI-based biplots were drawn to dissect the explained variation due to the GEI effect. The AMMI1 biplot represented the main effect of genotypes and environments on the IPCA1 score. Among the genotypes, G2, G6, G11, and G16 showed the highest IPCA1 scores and were therefore considered the least stable genotypes with large distances from the origin of the biplot; hence, these genotypes showed a high interaction with the test environments (Figure 1A).

The first four genotypes recommended for each test environment were identified using the AMM2 model. As shown in Table 3, genotype G2 ranked first in five environments (HAM1, HAM2, JOL2, ARK2, and MIN1) and appeared in eight out of sixteen environments in the top four rankings; genotype G20 ranked in eight out of sixteen environments in the top four rankings and was the first genotype in environment ARD1; genotype G4 ranked first rank in two environments (TAB1 and KAJ2) and appeared in the top four in other two environments (MAS2 and ARK2); genotype G18 was identified in the top four in four environments (TAB2, MAS2, MIN2, and JOL1); and genotype G15 was observed in the top four in six environments (MIN1, MIN2, KAJ1, ARD1, ARD2, and HAM1) and as dominant in the KAJ1 environment. Additional results are shown in Table 3.

The results for the estimated AMMI-based stability parameters along with the average grain yield for each genotype tested are shown in Table 4. Based on grain yield, the reference genotype (cv. Jolge), followed by G20, G2, G18, G3, and G4, showed the best average grain yield among the 16 test environments, while G10, G14, G17, G8, and G11 showed the lowest productivity. Genotype G20, followed by G14, G12, G17, and G6, showed higher stability compared to the other tested genotypes based on their low AMMI stability values (ASVs), average squared eigenvector values (EVs), AMMI-based stability parameter (ASTAB), the average sum between environments of the absolute value of GEI modeled using AMMI (AVAMGE), and IPCA point distance from the origin in space (Dz). According to the fitted AMMI model parameter (FA), genotypes G9, G12, G14, G17, and G20 were the most stable genotypes. The AMMI stability index (ASI) indicated that genotypes G20, G14, G12, G17, and G7 were the most stable genotypes. Based on the distance between the IPCA points and the origin in space (Da), genotypes G5, G9, G14, G17, and G20 showed the greatest stability compared to the other genotypes.

The results of the principal component analysis (PCA) showed that the first two PCAs with eigenvalues greater than 1 explained 90.68% of the total variation in grain yield and stability parameters. The biplot constructed using PCA1 and PCA2 was used to evaluate the correlation between stability parameters and grain yield (Figure 1B). Thus, the large cosine angle between the vectors of the two parameters showed a weak correlation, while the small cosine angle between these vectors showed a strong positive correlation. In contrast, no relationship was displayed at a cosine of 90°, while a strong negative correlation was displayed at a cosine of 180°. Based on the results, positive and strong correlations were observed between AVAMGE, FA, EV, DZ, DA, and ASTAB. On the other hand, grain yield was positively and strongly correlated with the ASV and ASI parameters. Therefore, the latter stability parameters can be expressed as a ‘dynamic’ stability concept.

### 2.3. GGE Biplots to Visualize GEI

**Figure 2:**
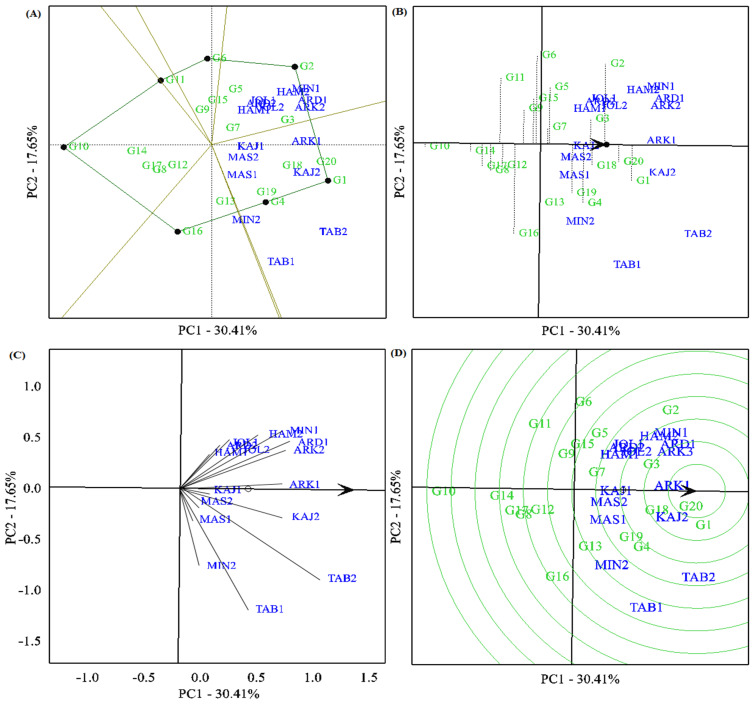
(**A**) The ‘which-won-where’ view of the GGE biplot showing mega-environments and their winning genotypes for grain yield. (**B**) Biplot for the simultaneous selection of grain yield and stability of the investigated barley genotypes. (**C**) The ‘discriminating power and representativeness’ view of the GGE biplot. (**D**) Comparison of promising genotypes of barley against the ‘ideal’ genotype for grain yield and stability across the 16 test environments.

The results of the GGE biplot analysis showed that the two PCAs accounted for 48.06% of the total grain yield variation in the different test environments. The polygon view of the GGE biplot clustered the test environments into two of the six sectors (Figure 2A). The environments of Tabriz (TAB1 and TAB2), Karaj (KAJ1 and KAJ2), and Mashhad (MAS1 and MAS2) along with MIN1 (Miandoab—first year) and ARK1 (Arak—first year) were placed in one sector and formed mega-environment I. The other environments, including Hamadan (HAM1 and HAM2), Joleh-Rokh (JOL1 and JOL2), Ardabil (ARD1 and ARD2), and the second-year data for Arak and Miandoab (ARK2 and MIN2), were considered mega-environment II. On the other hand, the best genotypes were G1 (reference genotype: Jolege), G2, G4, G6, G10, and G11. Among them, G16, G4, and G1 were the highest-yielding and most adapted genotypes for the first mega-environment. In contrast, the G2 genotype was specifically adapted to mega-environment II. Genotypes G6, G10, G11, and G16 showed no specific adaptation to any of the test environments. The “mean vs. stability” biplot viewpoint indicated that G1 (the reference genotype), followed by G20 and G2, had the highest average grain yield in the test environments. Genotypes G3, G14, G18, and G19 showed yields closest to the grand mean value due to their position in the biplot. The G20 genotype, with the high average grain yield, was the most stable, while G2, G4, and G19 showed significant yield variability in different environments. However, some genotypes, such as G10 and G14, with a low average yield, showed high stability (Figure 2B).

Figure 2C shows the discrimination power and representativeness of the test environments. Based on this biplot, test environments TAB1, TAB2, ARD1, MIN1, ARK1, and KAJ2 showed the highest value of discrimination power due to the large length of the environment vectors. Moreover, the representative ability of the test environments was determined using the average environment coordinate (AEC) and the angle between the test environment vectors. Accordingly, the ARK1, KAJ1, KAJ2, and MAS2 environments with smaller angles showed relatively strong representativeness, while MIN2, TAB1, ARD1, and JOL1 showed relatively weak representativeness. Therefore, KAJ2 and ARK1 were considered ideal environments due to their discrimination strength and representativeness potential. A comparative view of the GGE biplot was used to identify ideal genotypes (Figure 2D). According to the theory of Yan and Kang [20], an ideal genotype has the highest yield and stability in all test environments. Of the barley genotypes tested, genotypes G20, G1, G18, G3, G4, G19, and G2 were near the average environment axis (AEA) and were identified as ideal genotypes. Of these, genotype G20 was closest to the central circle in the biplot and showed specific adaptability to ARK1 and KRJ2 environments. However, genotypes G8, G10, G14, and G17 were found to be the most undesirable compared to other genotypes.

## 3. Discussion

**Figure 3:**
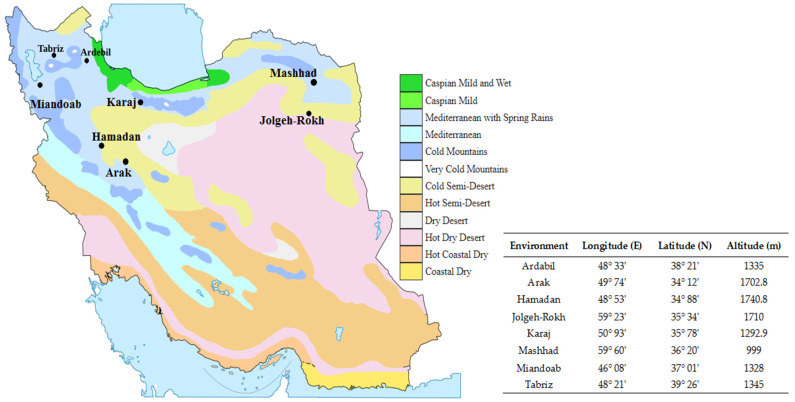
Agro-climatic zone map showing the test locations in this study (https://commons.wikimedia.org/wiki/File:Iran-climate-map-es.svg accessed on 10 January 2023).

In recent years, major parts of crop fields have been affected by various environmental stresses caused by climate change. These changes can have a negative impact on crop productivity and food security. Of the cereal crops, barley has a high tolerance to abiotic stresses, and this trait has led to its cultivation in highly variable weather environments and even in marginal crop fields. Multi-environment trials (METs) provide an opportunity to dissect GEI effects as well as identify target environments for economical seed production [29]. Moreover, these trials play a key role in identifying superior genotypes and determining their general and specific adaptations to different environments [30]. To obtain superior genotypes with high yield and stability, two strategies have been proposed: (i) dividing mega-environments into homogeneous regions to obtain varieties with specific adaptations and (ii) identifying genotypes with general adaptability in different environments [31]. Based on the literature, both strategies have been widely used to develop stable varieties with high performance [8,15,32,33,34,35,36]. In this study, several new barley genotypes were evaluated in 16 cold environments in Iran. As shown in Figure 3, some test environments are located in regions with cold semi-desert climates (e.g., Hamadan, Arak, Jolge-Rokh, and some parts in Arak); on the other hand, some environments (e.g., Tabriz, Ardabil, and Mashhad) have a wide range of weather conditions (from Mediterranean to cold). In addition, it is worth noting that the largest barley-growing areas in Iran are in cold regions. Therefore, planning breeding projects to identify and develop genotypes with specific and general adaptability is essential to achieve a high level of barley production in Iran.

The results of the AMMI analysis indicated that the effects of the environment (E) and the genotype-by-environment interaction (GEI) accounted for a greater proportion of the total variability than the effect of genotype (Table 2). In line with our results, previous studies have also shown that the environment was the main source of variation in barley samples under different environmental conditions [6,13,15,27,35]. The magnitude of the total sum of squares (TSS) for the GEI effect indicated significant differences in the genotypic response of barley to environmental conditions in cold regions in Iran. Therefore, the adaptation patterns and stability of the genotypes studied were analyzed using different methods. The AMMI1 biplot provided an opportunity to identify high-yielding and stable genotypes due to the distribution of genotypes based on the IPCA1 results and average grain yield. Based on this biplot, the G20, G14, and G10 genotypes, with high average yield relative to the overall average yield and the lowest IPC1 scores, were found to have high yield stability (Figure 1A). Moreover, this result was confirmed using the AMMI2 model, in which the G2 and G20 genotypes were found to be in the top four genotypes in eight of the sixteen test environments (Table 3). The results for the AMMI-based stability parameters showed that genotype G20, with an average grain yield of 7.59 tons ha^−^^1^, was the most stable genotype compared to the others. Using another AMMI statistic, we found that the ASV and ASI positively and significantly correlated with grain yield, which in turn revealed a ‘dynamic’ concept of stability (Figure 1B). Consequently, barley breeders can use these parameters to select genotypes that have a stable yield similar to the average yield of all genotypes in the tested environments [37].

The biplot of polygons (Figure 2A) classified the test environments into two mega-environments. The first mega-environments included Tabriz (TAB1 and TAB2), Karaj (KAJ1 and KAJ2), and Mashhad (MAS1 and MAS2) along with MIN1 (Miandoab—first year) and ARK1 (Arak—first year), for which genotypes G1 and G4 were identified as the best-adapted genotypes. In addition, genotype G20, with acceptable yield and yield stability, was placed in this mega-environment. The Hamadan (HAM1 and HAM2), Jolgeh-Rokh (JOL1 and JOL2), and Ardabil (ARD1 and ARD2) test environments and the second-year data for Arak and Miandoab (ARK2 and MIN2, respectively) formed the second mega-environment, where the G2 genotype was identified as the best adapted to them. Overall, the clustering of test environments followed the climatic clustering scheme (Supplementary Figure S1). The results of the cluster analysis based on temperature data (minimum, maximum, and average) grouped all test environments into two main clusters. The first cluster included Hamadan, Jolgeh-Rokh, and Ardabil, while the second consisted of Mashhad, Karaj, Arak, and Tabriz. These results were consistent with the results of the mega-environments clustering, so it can be concluded that the tested genotypes can specifically respond to environmental changes. In other words, the obtained mega-environment pattern in this study was related to the classification obtained from the weather data, and this finding was confirmed using the Mantel test (r = 0.548 **) (Figure S2). Moreover, these results were further supported with the AMMI model, in which fourth-order genotypes were identified in target environments. For example, G2 was identified as a high-yielding genotype in the Karaj and Mashhad environments, while G2 was selected for the Hamadan and Jolgeh-Rokh environments (Table 3). Therefore, these results may confirm the possibility of two microclimates in the cold regions in Iran. However, this prediction requires further research using other data sets from different years as well as different field crops.

These results were further supported by the freezing test. As shown in Table 1, genotype G4 appeared to be the most tolerant and produced significant grain yield under field conditions (freezing test at Karaj station) compared to the other genotypes. Therefore, this genotype can be recommended for cultivation in target environments (Tabriz, Mashhad, and Karaj). In addition, the G2 genotype, with acceptable cold tolerance, was suitable for cultivation in the second mega-environment. Since the target environments belonging to this mega-environment are located in semi-arid regions, it is recommended to evaluate the response of this genotype to terminal drought stress.

According to Yan [38], the environment with the most discriminating and representative power can be considered the ideal target environment for evaluating new varieties for their full yield potential. According to this theory, test environments can be divided into three types. Type I includes environments that provide little information on genotypes due to their short vectors; therefore, they should not be used as test environments. Type II includes environments that are suitable for identifying the best high-yielding genotypes due to their small angles with the AEC and long vectors. Type III consists of environments that are useful for eliminating unstable genotypes due to their large angles with AECs and long vectors. In this study, the KAJ2 and ARK1 environments had high discriminating power and representativeness potential. Therefore, these environments can be recommended as desirable environments (Type II) for testing advanced genotypes for yield and yield stability during early breeding programs in cold regions in Iran. On the other hand, among the test locations, the Mashhad location (MAS1 and MAS2) was a Type I environment; therefore, this location can be omitted from stability and adaptability experiments to reduce the cost of field evaluations (Figure 2C). Another result of the GGE biplot was a positive and significant correlation between the Hamadan (HAM1 and HAM2), Jolgeh-Rokh (JOL1 and JOL2), and Arak (ARK1 and ARK2) environments, suggesting that two of the three locations can be removed from barley METs for cold regions in Iran. Another comparison view of the GGE biplot identified the ideal genotypes. Theoretically, genotypes near the beginning of the biplot may have broader adaptation and could be identified as ideal genotypes [39]. Based on this biplot, genotype G20, followed by G1, G18, G3, G4, G19, and G2 were placed closest to the AEA cutoff and selected as ideal genotypes. Of these, the G20 genotype with good correlation in ARK1 and KAJ2 environments showed specific adaptability to these environments (Figure 2D). In summary, our results revealed that both AMMI and GGE models deciphered a similar pattern for determining high-yielding and stable barley genotypes. Therefore, the relative contribution of both models to the identification of superior genotypes in this study was consistent with previous studies on barley and other crops [13,26,27,40,41].

Genotypes that are best suited to specific environmental conditions can be detected using AMMI analyses, which can estimate the effect of the genotype interaction in each environment. For yield, a significant GEI was demonstrated using an AMMI analysis. The high stability of genotypes is related to the AMMI stability value. Determination of the main effect of the genotype, environment, and the most significant GEIs can be completed using AMMI results displayed on the GE biplot. AMMI models are able to measure the importance of environments, genotypes, and their interactions using a value that measures genotype stability across all environments, given the yield [42]. Genotype G20, with the high average yield relative to the overall average yield and the lowest IPC1 score, was shown to have high yield stability and thus is recommended for inclusion in breeding programs.

## 4. Materials and Methods

### 4.1. Plant Materials, Field Layout, and Experimental Design

A set of eighteen promising barley genotypes, along with two introduced varieties (Jolge (G1) and Mahtab (G19)) used as reference genotypes, were studied using multi-environment trials (Table 5). Field evaluations were carried out at eight cold-weather test stations (including Ardabil, Miandoab, Arak, Hamadan, Mashhad, Jolge Rokh, Tabriz, and Karaj) in Iran for two consecutive growing seasons (2019–20/2020–21) (Figure 3). Additional information on geographical and climatic data is presented in Supplementary Table S1. In all test locations, field trials were conducted using a randomized complete block design (RCBD) in three replications. The experimental plots consisted of six rows, each 6 m in length with 20 cm spacing between rows. The sowing density in each plot was 400 seeds per m^2^. Sowing was carried out using an experimental planter (Wintersteiger, Ried, Austria). At each station, basic fertilizers such as N and P_2_O_5_ were applied at 32 and 100 kg ha^−^^1^, respectively, before sowing. During the growing season, five irrigations were applied at growth stages 00, 32, 51, 75, and 85 Zadoks’ [43] at all test locations. In addition, 40 kg ha^−^^1^ of N was again applied at the stem elongation stage (ZGS 31). At harvest time (based on the time of physiological maturity at each test station), a combine harvester (Wintersteiger, Ried, Austria) was used to harvest the experimental plots. Grain yield was determined for each genotype at the test locations and used for further statistical analysis.

### 4.2. Evaluation of the Lethal Temperature (LT_50_) for Genotypes

All genotypes were tested for cold hardiness under field conditions and in a programmable freezer. The field experiment was performed at the Cereal Research Department, Seed and Plant Improvement Institute (SPII) Karaj, Iran (longitude 35° N, latitude 51° N, and altitude of 1132 m above sea level), which is characterized by a moderately cool temperate region. Plant materials were arranged in a randomized complete block design (RCBD) with two replicates. Experimental plots consisted of six rows, each 6 m long, with 20 cm spacing between rows. The seed density in each plot was 450 seeds per m^2^. Seeds were sown using an experimental planter (Wintersteiger, Ried, Austria) on 3 November 2018. After germination and early growth under field conditions, cold-acclimated seedlings were sampled for freeze testing during the first week of January 2019. Freeze tests to estimate the LT_50_ (the temperature at which 50% of the plants perish due to cold weather) were performed as described by Limin and Fowler [44]. Plant crowns were planted in pots containing moist sand and transferred to a programmable freezer, which was maintained at −3° C for 12 h. The freezing temperature was lowered in a constant gradient of −2 °C per hour until it reached −21 °C. At 2 °C intervals for each of the ten test temperatures (from −3 °C to −21 °C), five plants from each genotype from both replications were selected and transferred to the outside of the freezer. The crowns of the plants were replanted, and LT_50_ was recorded based on regrowth after 21 days under controlled glasshouse conditions. At harvest time, the remaining experimental plots for each genotype were harvested under field conditions using an experimental combine harvester (Wintersteiger, Ried, Austria). Finally, thousand-grain weight and grain yield were recorded for each genotype.

### 4.3. Statistical Methods

Grain yield data obtained from the 16 test environments (a combination of 2 cropping seasons and 8 test locations) were subjected to AMMI analysis. Several AMMI-based stability indices were used to determine the phenotypic adaptability and stability of the barley genotypes tested. To assess the relationships between the estimated stability indices and grain yield, a principal component analysis (PCA) was performed. In addition, grain yield data were graphically analyzed using a GGE biplot model. The biplots were rendered based on the first two principal components (PCAs) obtained using singular value decomposition (SVD). All statistical analyses were calculated using the packages ‘pheatmp’ [45], ‘metan’ [46], ‘FactoMineR’ [47], and ‘factoextra’ [48] in R software [49]. In addition, a cluster analysis based on Ward’s algorithm was conducted to group the test environments using weather data. To examine the relationship between the clustering pattern in the environment and the identified mega-environments, a Mantel test was calculated using XLSTAT software (XLSTAT, Addisonsoft, Paris, France).

## 5. Conclusions

The collective analysis using the AMMI and GGE biplot models identified two mega-environments for barley-growing cold regions in Iran, which in turn provided important implications for future barley breeding programs. Moreover, the promising new genotypes examined in this study should be recommended for each mega-environment to improve productivity and stability. When considering the results obtained in this study, the high-yielding and stable genotypes in each mega-environment were generally different, which in turn was in accordance with a recommendation from Gauch [22], who noted that genotype stability is a meaningful goal only in each mega-environment and not in multiple mega-environments. In conclusion, our results showed that the G2 and G20 genotypes can be recommended as newly promising barley genotypes for further investigation before commercial release.

## Figures and Tables

**Table 1:** The response of the evaluated barley genotypes to the freezing test under field conditions at the Karaj research station during the 2019–2020 cropping season.

Genotype Code	LT_50_ (°C)	Thousand-Grain Weight (g)	Grain Yield (Tons ha^−1^)
G1	−8	40.20	8.71
G2	−8	37.80	5.27
G3	−8	39.00	7.01
G4	−10	38.80	7.62
G5	−8	38.80	7.87
G6	−7.5	41.30	6.20
G7	−7	34.50	8.32
G8	−8	42.00	6.90
G9	−7.5	42.50	7.02
G10	−8	35.90	5.94
G11	−8	39.40	7.33
G12	−6	42.60	6.56
G13	−7.5	39.30	6.69
G14	−8	40.40	7.06
G15	−6	37.90	6.44
G16	−6.5	45.20	7.10
G17	−6	46.60	7.04
G18	−8.5	35.90	7.21
G19	−7	43.10	7.86
G20	−8	41.50	8.51
LSD (0.05)	3.68	6.46	2.02

**Table 2:** Result of the AMMI analysis of grain yield data for the evaluated barley genotypes across 16 test environments in cold regions in Iran.

Source	df	SS	MS	*F*-Value	Probability	% TSS
Total	959	1972.3	2.057			
Treatments	319	1400	4.389	5.61	**	
Genotype (G)	19	98.4	5.18	6.62	**	4.99
Environment (E)	15	975.2	65.011	21.56	**	49.44
Replication	32	96.5	3.015	3.85	**	
GEI	285	326.4	1.145	1.46	**	16.55
IPCA1	33	75	2.273	2.9	**	22.98
IPCA2	31	59.4	1.916	2.45	**	18.20
IPCA3	29	44	1.516	1.94	**	13.48
IPCA4	27	37.5	1.388	1.77	**	11.49
IPCA5	25	30	1.2	1.53	*	9.19
Residuals	140	80.6	0.576	0.74		
Error	608	475.9	0.783			

* and ** Significant at *p* < 0.05 and *p* < 0.01, respectively. TSS indicates the total sum of squares.

**Table 3:** The first four genotypes of barley selected using the AMMI model in each environment.

Environment	Mean	IPCA1 Score	1	2	3	4
ARD1	7.86	−0.4858	G20	G15	G2	G1
ARD2	6.212	−0.4278	G11	G15	G2	G13
ARK1	7.558	−0.0473	G7	G20	G8	G2
ARK2	5.579	−0.4088	G2	G3	G1	G4
HAM1	7.289	−0.3568	G2	G6	G3	G9
HAM2	10.014	−0.5616	G2	G1	G15	G11
JOL1	6.244	−0.4681	G6	G3	G10	G18
JOL2	6.838	−0.3916	G2	G6	G7	G3
KAJ1	7.306	0.0286	G15	G13	G20	G12
KAJ2	6.614	0.2851	G4	G1	G20	G19
MAS1	6.85	0.3332	G7	G20	G16	G13
MAS2	6.379	0.1469	G18	G1	G20	G4
MIN1	6.827	−0.6028	G2	G15	G20	G5
MIN2	6.546	0.8114	G16	G13	G15	G18
TAB1	7.46	1.2446	G4	G13	G19	G1
TAB2	8.393	0.9007	G1	G18	G16	G20

ARD, ARK, HAM, MIN, MAS, KAJ, JOL, and TAB indicate Ardabil, Arak, Hamadan, Jolgeh-Rokh, Karaj, Mashhad, Miandoab, and Tabriz locations, respectively. Numbers 1 and 2 indicate the first and second cropping years, respectively.

**Table 4:** The results for the estimated AMMI-based stability parameter along with grain yield for the investigated barley genotypes.

Genotype Code	GY	ASTAB	ASI	ASV	AVAMGE	DA	DZ	EV	FA
G1	7.63	1.330	0.136	0.745	7.190	2.260	0.594	0.071	5.110
G2	7.49	0.990	0.193	1.060	7.250	2.140	0.467	0.043	4.570
G3	7.39	0.510	0.105	0.576	4.550	1.470	0.353	0.025	2.160
G4	7.39	1.440	0.153	0.841	6.620	2.380	0.612	0.075	5.670
G5	7.11	0.620	0.136	0.745	5.220	1.670	0.377	0.028	2.770
G6	7.1	1.310	0.218	1.200	7.740	2.410	0.552	0.061	5.810
G7	7.18	1.010	0.083	0.457	5.760	1.890	0.543	0.059	3.570
G8	6.77	0.790	0.084	0.464	5.090	1.700	0.473	0.044	2.880
G9	7.08	0.480	0.084	0.463	4.510	1.380	0.351	0.025	1.910
G10	6.41	1.840	0.101	0.556	8.060	2.640	0.700	0.098	6.960
G11	6.93	1.660	0.194	1.070	8.120	2.610	0.646	0.083	6.820
G12	6.98	0.410	0.071	0.392	3.920	1.280	0.323	0.021	1.630
G13	7.22	1.440	0.231	1.270	8.230	2.570	0.563	0.063	6.600
G14	6.64	0.340	0.047	0.258	3.600	1.120	0.304	0.018	1.250
G15	7.16	1.770	0.112	0.613	8.810	2.600	0.686	0.094	6.760
G16	6.97	1.540	0.242	1.330	8.990	2.660	0.584	0.068	7.070
G17	6.69	0.380	0.072	0.395	3.910	1.220	0.316	0.020	1.490
G18	7.43	1.310	0.185	1.020	6.910	2.360	0.558	0.062	5.580
G19	7.33	0.420	0.117	0.643	4.290	1.390	0.308	0.019	1.920
G20	7.59	0.380	0.039	0.214	3.580	1.190	0.321	0.021	1.430

GY, ASTAB, ASI, ASV, AVAMGE, DA, DZ, EV, and FA indicate grain yield, AMMI-based stability parameter, AMMI stability index, AMMI stability value, average sum across environments of the absolute value of GEI modeled using AMMI, distance between IPCA points and the origin in space (A and Z), average of the squared eigenvector value, and fitted AMMI model, respectively.

**Table 5:** The pedigree of the 18 evaluated, newly promised barley genotypes along with the reference genotype across 8 locations during 2 years in the cold agro-climate in Iran.

Genotype Code	Line/Pedigree	Spike Type	Growth Type
G1	Jolge (Reference 1)	Six-row	Winter
G2	Bahman/3/Makouee//Zarjow/80-5151	Six-row	Winter
G3	Alger//CI10117/Choyo/3/Makouee/4/STB-12	Six-row	Winter
G4	Comp.Cr229//As46/Pro/3/Srs/4/Express/5/Goharan/6/Goharan	Six-row	Facultative
G5	Zarjow/80-5151//Makouee/3/Makouee	Six-row	Winter
G6	Makouee//Zarjow/80-5151/3/Bahman	Six-row	Winter
G7	Radical/Birgit//Pamir-154/3/Goharan	Six-row	Facultative
G8	Cali92/Robust//ND16301	Two-row	Spring
G9	Radical/Birgit//Pamir-154/3/Goharan	Six-row	Facultative
G10	Yousef/4/82S:510/3/Arinar/Aths//DS 29	Six-row	Facultative
G11	Courlis/Rhn-03//Karoon	Six-row	Spring
G12	Mahtab/Goharan	Six-row	Spring
G13	Comp.Cr229//As46/Pro/3/Srs/4/Express/5/Goharan/6/Goharan	Six-row	Spring
G14	Pamir-147/Sonata/8/Alpha/Durra/7/P101/5/3896/1-15/3/3896/28//584/28/4/5050/6/Tipper	Two-row	Winter
G15	Courlis/Rhn-03//Karoon	Six-row	Winter
G16	Bda/Rhn-03//ICB-107766/3/Yousef	Six-row	Facultative
G17	Sonata/8/Api/CM67//Hma-03/4/Cq/Cm//Apm/3/RM1508/5/Attiki/6/Aths/7/SP(6H) /Apro//Ca1Mr/3/ROD586/Apm/4/Aths/9/Sararood	Two-row	Winter
G18	Nadawa/Rhn-03//Birka	Six-row	Spring
G19	Mahtab (Reference 2)	Six-row	Facultative
G20	Bahman/3/Alger//CI10117/Choyo	Six-row	Winter

## Data Availability

The data in this manuscript are available from the corresponding authors upon reasonable request.

## Supplementary Materials

The following supporting information can be downloaded at: https://www.mdpi.com/article/10.3390/plants12132410/s1, Table S1. Agro-climatic characteristics of the test environments during the 2019–2021 cropping years in the cold regions in Iran; Figure S1. The grouping pattern in the test environments based on weather data during the 2019–2021 cropping years in the cold regions in Iran. ARD, ARK, HAM, MIN, MAS, KAJ, JOL, and TAB indicate Ardabil, Arak, Hamadan, Jolgeh-Rokh, Karaj, Mashhad, Miandoab, and Tabriz locations, respectively. Numbers 1 and 2 indicate the first and second cropping years, respectively; Figure S2. Mantel’s test results between the estimated proximity matrices (Euclidean distance) for the grouping patterns in the test environments based on weather data and grain yield.
