# Supplementary material for: Selection of High-Yielding and Stable Genotypes of Barley for the Cold Climate in Iran
Source: Plants (Basel). 2023 Jun 22;12(13):2410. doi: 10.3390/plants12132410 (PMC10346509; doi:10.3390/plants12132410)
Supplement: Supplementary file 1 [file plants-12-02410-s001.zip › plants-2447534-supplementary.pdf]

plants

MDPI

Table S1. Agro-climatic characteristics of the test environments during the 2019-2021 cropping years in the cold regions of Iran.

Cropping year	Location	Minimum Temperature (°C)										
		Sep	Oct	Nov	Dec	Jan	Feb	Mar	Apr	May	Jun	Jul
2019-2020	Arak	6.6	-2.4	-3.8	-6.6	-13.8	-4.2	0	5	10.8	15.2	17
	Jolgeh-	-2	-3	-3	-8.4	-12.4	-4.2	-0.8	5.6	7.8	12.2	8.2
	Ardabil	1	-5	-8	-9.5	-14	-7.6	-4.4	-0.6	3.2	7.6	8.4
	Mashhad	5.3	-2.2	-3.8	-12	-4.6	-3	-0.8	9.2	14.2	16	16.9
	Tabriz	5	-5.2	-4.2	-6.6	-13.8	-4.2	-0.9	5.8	8.5	15	15.7
	Miandoab	4.8	-5	-5	-10.6	-16	-4.4	-1.4	3.8	6	11	11
	Karaj	9.5	-2	-0.7	-4.9	-10.4	-2.3	0	5.6	12.1	14.3	14.9
	Hamadan	3	-8.5	-10.9	-12	-15.9	-7.2	-3.8	2	6.9	8.3	11.9
2020-2021	Arak	1.4	-0.6	-3.4	-8.2	-8.8	-4.2	-2	6.6	11.4	17.2	16.4
	Jolgeh-	-1	-7	-7	-14	-11	-4	-3	4	5	12	7
	Ardabil	-2	0	-17.2	-14.6	-20.8	-4.2	-4.9	2.2	8.2	7.8	20.7
	Mashhad	1.4	-4.3	-7.2	-8.6	-8.3	-7.8	1.5	11.4	15.6	17.4	15.2
	Tabriz	5.4	0.8	-3.8	-12.2	-13.2	-4.2	-1	8.2	10.60	15.4	11.9
	Miandoab	3.6	0	-5.2	-9.6	-12.2	-7.4	-3.40	3.70	7.60	11.70	12.10
	Karaj	5	2.4	-3.4	-6.9	-7.4	-5	-1.40	8.50	11.10	15.60	16.80
	Hamadan	-1.7	-3.6	-8.5	-11.2	-12.3	-8.8	-6.60	2.80	6.00	11.40	12.00
		Maximum Temperature (°C)										
2019-2020	Arak	33.4	19.8	15.6	14	16.8	20.8	22.40	29.60	36.80	37.40	39.60
	Jolgeh-	26.8	24.8	16	15.2	9.8	18.2	22.8	28.6	35	35	36.8
	Ardabil	32.4	20.2	16.4	15.2	13	20	21.4	26.2	34.2	39	38.4
	Mashhad	37.2	18.6	18	21.8	22.6	25.2	24.1	33.7	40.1	41.4	39.4
	Tabriz	30.2	18.4	13.8	15.4	11.4	19.2	21.7	27.2	34.8	40.4	38
	Miandoab	31	18.2	16.6	13	13	19	22.4	27	32.6	38	38.8
	Karaj	35.1	18.9	14.7	14	18.8	20.9	20.9	28.4	37.5	38.6	40.1
	Hamadan	30.8	17.5	14.1	10.1	14.8	20	29.2	35.1	42.3	36.6	38.1
2020-2021	Arak	32.8	25	15.6	16.6	20.2	21.8	27	33.4	38.2	38.4	39.8
	Jolgeh-	24	19	15	8	12	21	27	28	31	35	33
	Ardabil	26.2	27	11.2	15.2	19.5	20.2	29.6	31.9	34.6	38.4	39.8
	Mashhad	27.7	28.6	19.6	23.1	26	24	32.6	35.7	41.5	43.2	39.7
	Tabriz	31.4	24.4	10.5	13	19.2	19.1	24.6	33.2	38.3	39.8	38.4
	Miandoab	28.6	23.4	10.4	13.9	18.7	19.1	24.8	32.6	35	38.1	36.1
	Karaj	31.9	24.6	12.6	14	19.8	21.6	28.1	34.5	39.3	40.8	40
	Hamadan	30.7	24.4	11.6	17	17.9	20.2	26.9	32.4	39	37.3	38.7
		Average Temperature (°C)										
2019-2020	Arak	19.3	7.8	3.9	1.7	2	8.1	10.4	16.9	24.4	26.4	27.8
	Jolgeh-	12.4	10.9	6.5	3.4	-1.3	7	11	17.1	21.4	23.6	22.5
	Ardabil	14.2	6.2	3.3	1.4	-1.3	5.8	6.9	12.9	18.9	19.2	18.4
	Mashhad	18.6	9.5	5.8	3.9	3.9	9.8	10.9	19.5	26.7	27.8	27.8
	Tabriz	18.2	8.3	3.8	0	0	6.7	9.6	15.5	23	26.1	26.3
	Miandoab	15.7	6.9	2.6	-0.7	-2.6	5.9	9.3	14.6	20.5	23.3	23.6
	Karaj	19.5	9.6	6	3.9	3.9	10	10.2	17.2	25.4	26.5	27.2
	Hamadan	16.9	5.8	1.4	0	0	6.1	8	14.2	20.5	23.5	24.9

2020-2021	Arak	16.6	11.3	4.3	2.3	6	7.5	14.3	19.3	24.8	18.4	8.9
	Jolgeh-	11.5	6	4	-3	0.5	8.5	12	16	18	23.5	20
	Ardabil	12.1	10.1	11	0.6	5.4	1.6	10.9	15.1	19.2	12	7.2
	Mashhad	15	19.6	3.8	4.5	9	8.3	16.6	21.7	28.3	16.5	9.3
	Tabriz	17.3	11.6	2.7	-1.2	2.8	4.6	11.9	19	24.3	16.8	9.1
	Miandoab	15.4	10.5	2.5	0	8.8	4.5	11.2	17.8	21.5	14.7	7.9
	Karaj	17.8	13.8	4.5	3.5	7.9	7.8	15.4	21.3	26.6	18.3	10.3
	Hamadan	14.3	11.6	2.4	0.5	3.9	4.8	11	16.5	21	15.3	7.1
							Precipitation (mm)					
2019-2020	Arak	20.5	47.9	40.3	24	16.2	104.9	125.3	26.9	0.5	0	1.8
	Jolgeh-	1.1	62.9	7.6	21	35.4	76.1	48.7	39.2	10.8	0	0
	Ardabil	51.9	19.1	3.1	22.7	25.7	15.6	27.5	56.5	6.6	4.3	4.8
	Mashhad	2.3	17.9	8.4	41.7	9.3	26.7	141.5	37.1	0.5	3.7	1.4
	Tabriz	21.4	3.5	10	41.7	16.1	33.6	60.8	41.2	11.6	17.6	3.5
	Miandoab	24.4	1.4	45.8	47.4	48.8	48.1	56.9	52.1	8.1	8.3	0
	Karaj	4.9	103.6	30.2	38.2	16.6	117.5	94.4	53.7	2	0	0.8
	Hamadan	15.8	38.1	55.6	13.6	10.8	97.9	115.6	41.3	4	0	0
2020-2021	Arak	0.6	8.7	87.5	0.7	28.3	41.6	1.7	20	0.9	0	1.6
	Jolgeh-	0	8	5	54	12	33	24	65	6	0	0
	Ardabil	30.3	36.1	52.4	25.4	24.3	26.8	5.5	16.8	6.3	3.5	11.8
	Mashhad	0	12.1	26.5	4.6	0	28.9	6	16.5	0	1.3	0
	Tabriz	16.4	54.3	21.7	16.8	52.7	12.4	27.5	49.4	6.6	14.2	21.9
	Miandoab	0	53.5	33.2	3.3	42.8	29.7	45.1	20.4	0.2	5.5	10.6
	Karaj	9.7	18.4	99.9	25.3	56.1	23	0.4	15.4	9.1	2	2.1
	Hamadan	6	12	106.7	2.2	75.4	113.7	6.6	7.3	1.6	0	1.8

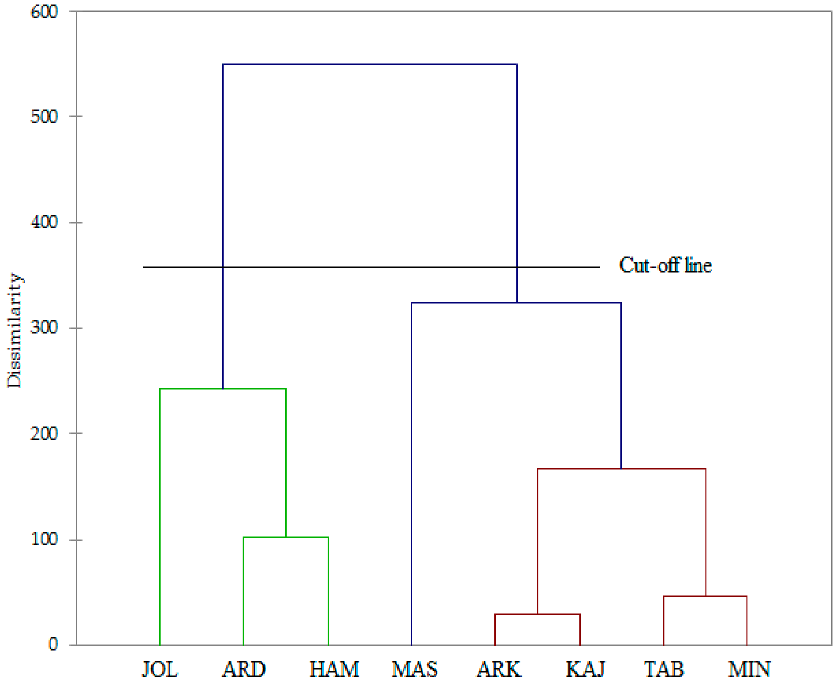

Figure S1. The grouping pattern of test environemnt based on weather data during the 2019-2021 cropping years in the cold regions of Iran. ARD, ARK, HAM, MIN, MAS, KAJ, JOL, and TAB indicate Ardabil, Arak, Hamadan, Jolgeh-Rokh, Karaj, Mashhad, Miandoab, and Tabriz locations, respectively. Numbers 1 and 2 indicate the first and second cropping year, respectively.

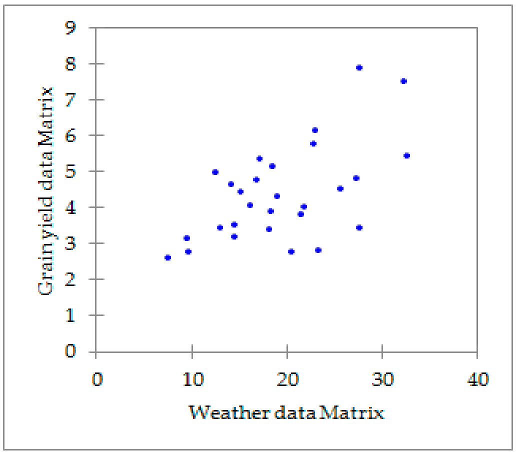

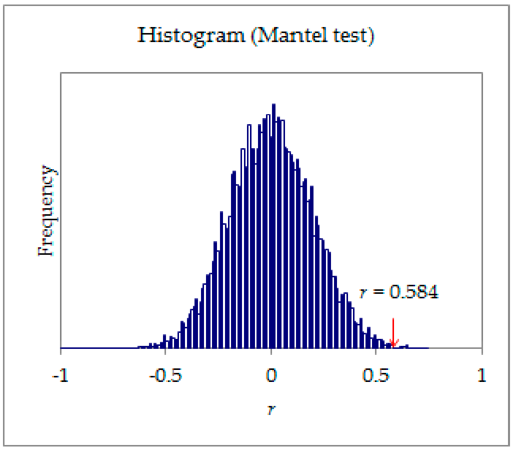

Figure S2. The Mantel's test between estimated proximity matrices (Euclidean distance) for the grouping patterns of the test environments based on weather data and grain yield.
